# Supplementary material for: Systematic Physical Exercise and Spirulina maxima Supplementation Improve Body Composition, Cardiorespiratory Fitness, and Blood Lipid Profile: Correlations of a Randomized Double-Blind Controlled Trial
Source: Antioxidants (Basel). 2019 Oct 23;8(11):507. doi: 10.3390/antiox8110507 (PMC6912262; doi:10.3390/antiox8110507)

**Supplementary File 3.** Statistical Analysis

**Table 2**

**Descriptive statistics**


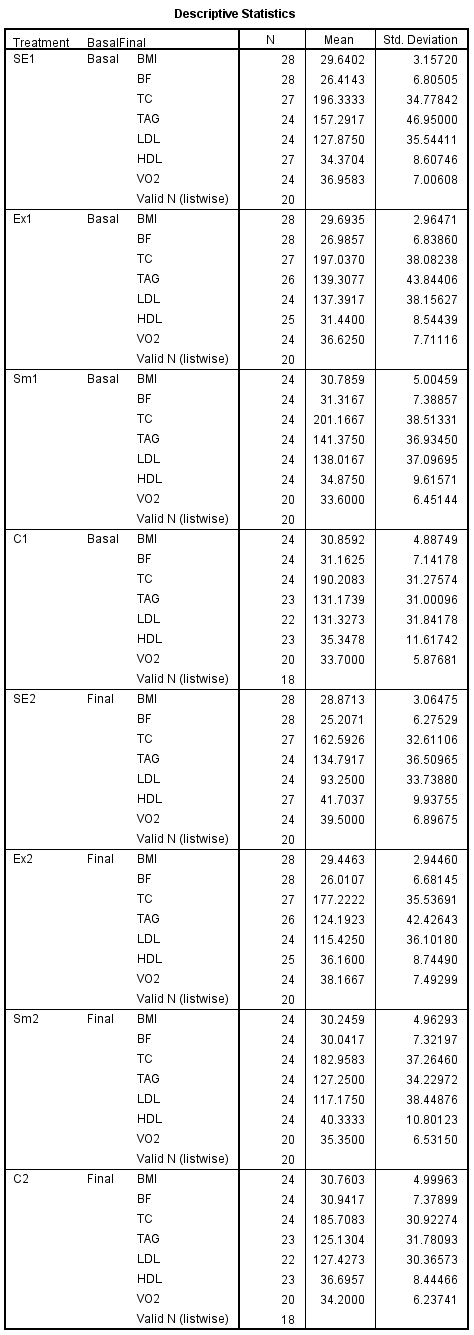


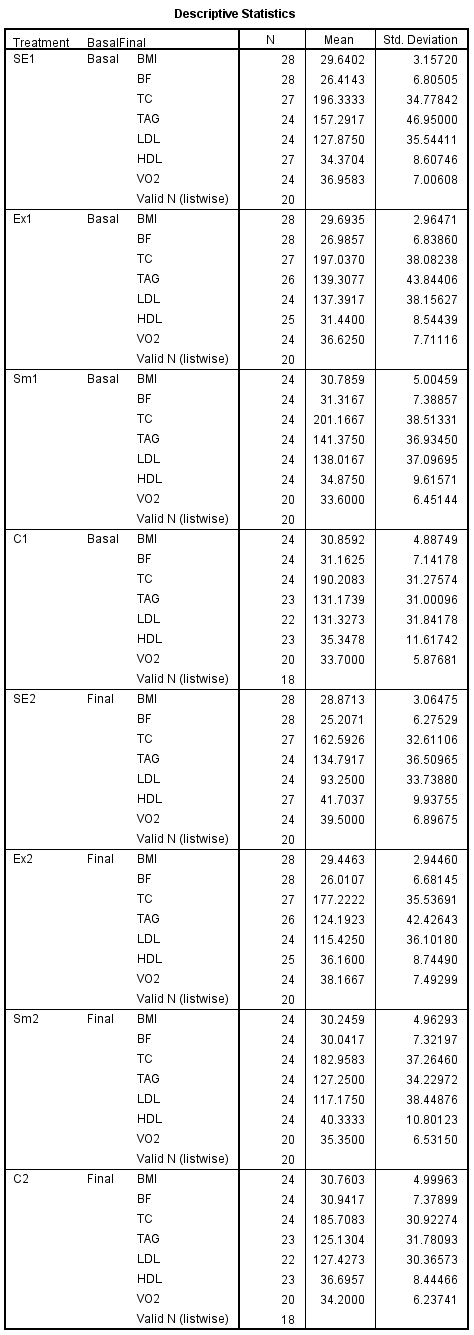

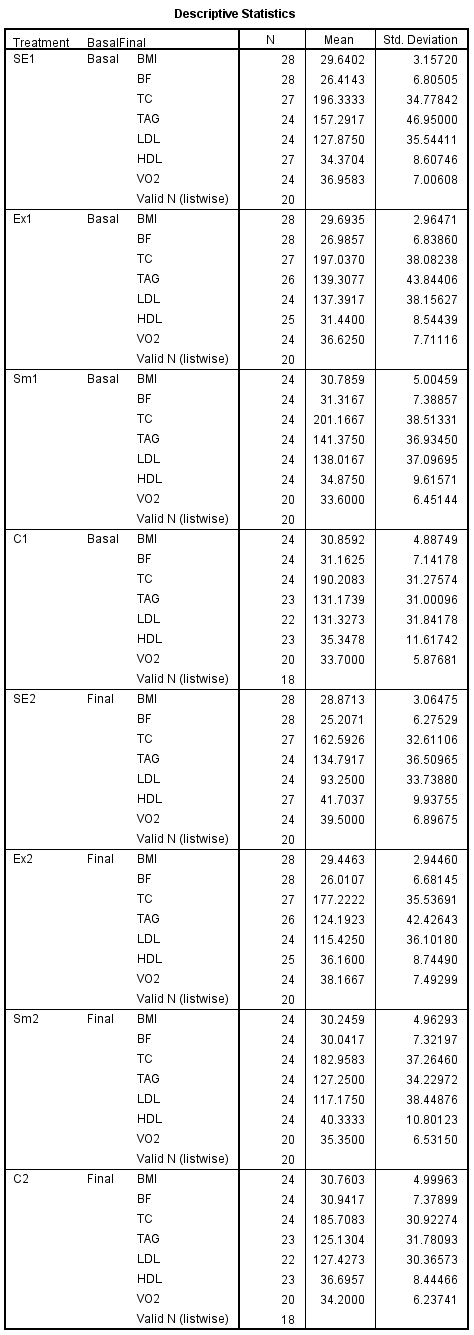


**Paired *t* test**

Exercise and *Spirulina maxima* treatment


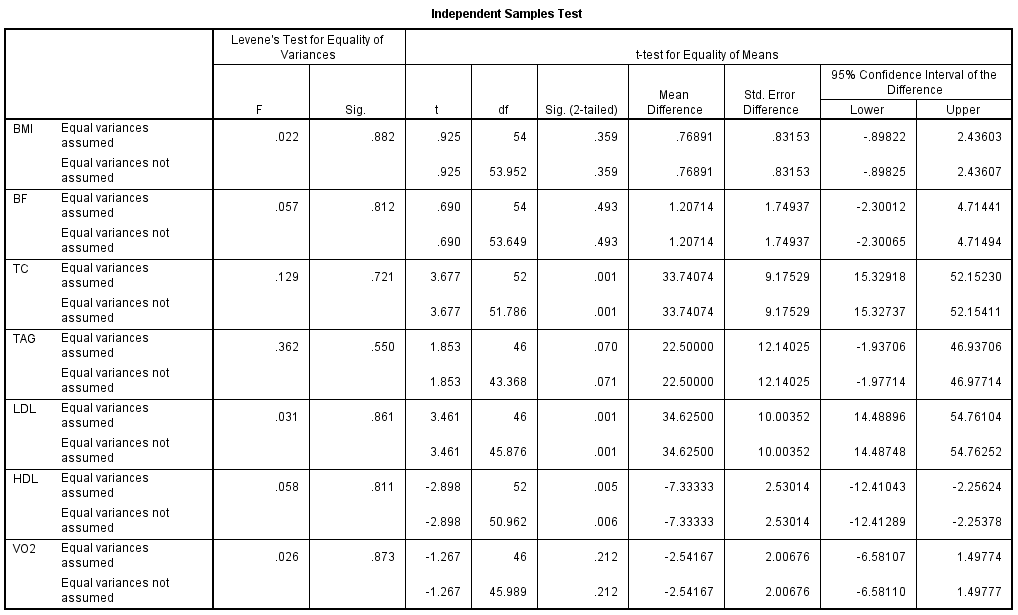


Exercise and placebo treatment


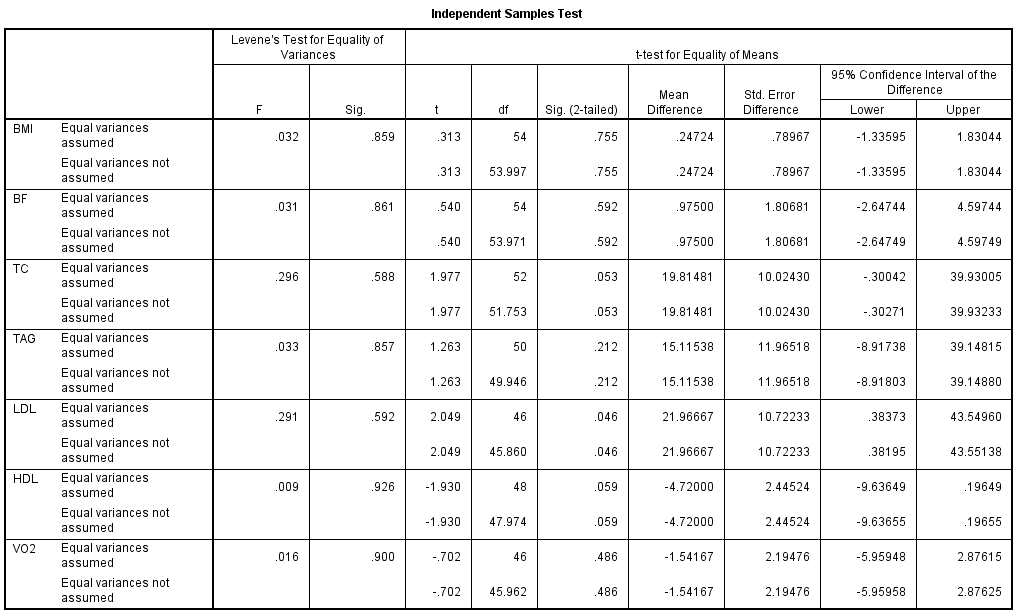


*Spirulina maxima* without exercise treatment

**
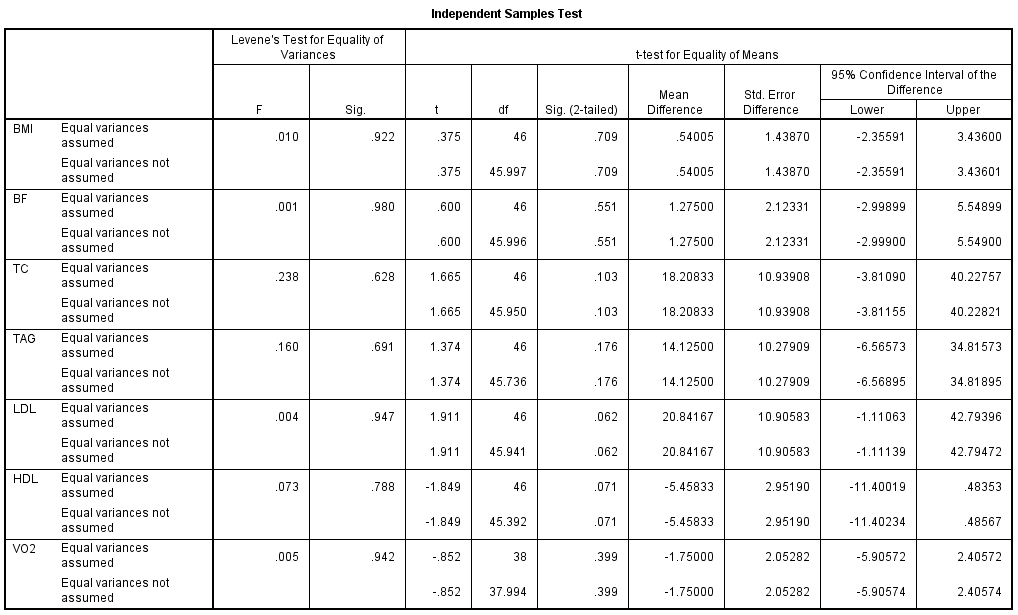
**

Placebo without exercise treatment (Control)

**
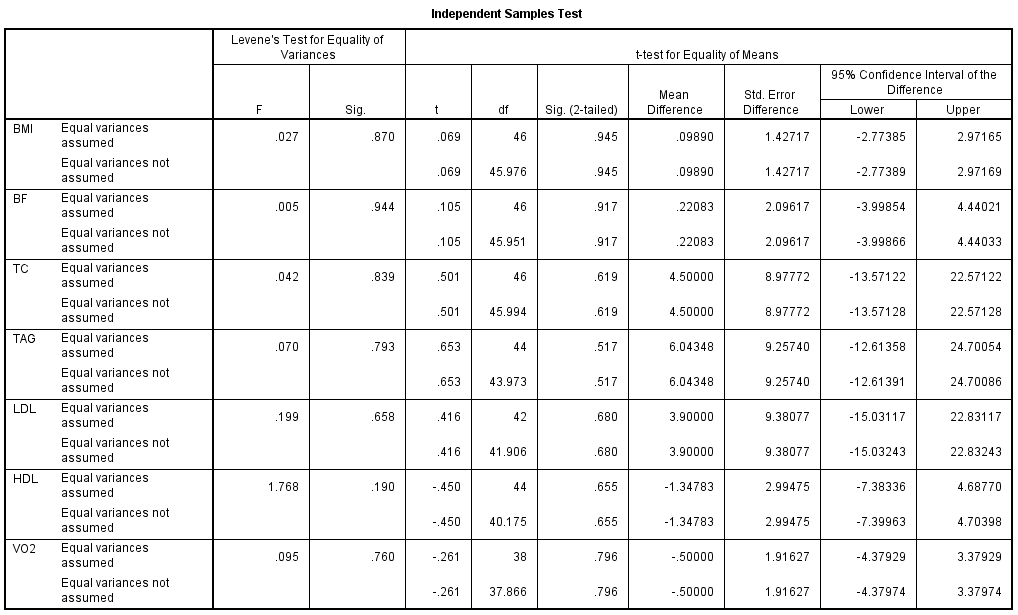
**

Dyslipidemic obese subjects

**
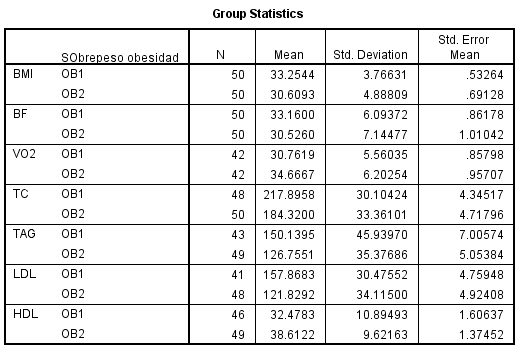
**

**
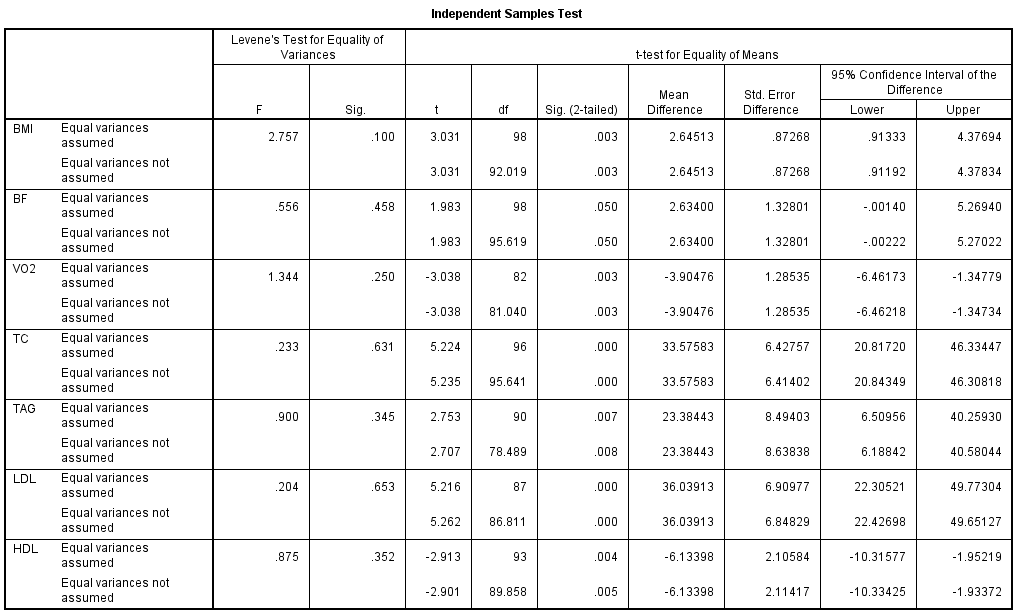
**

**Table 3**

Spearman’s correalation


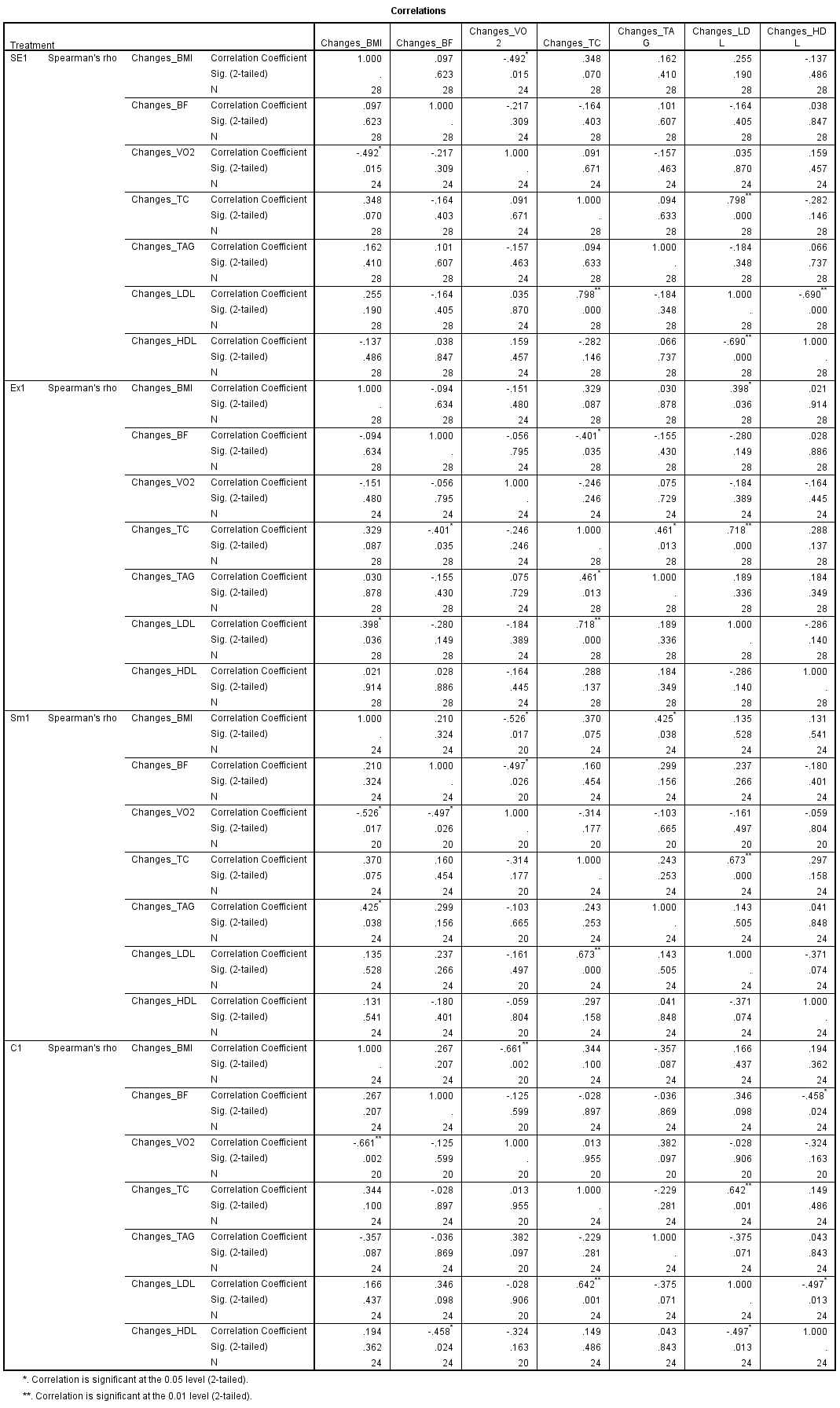

Supplement: Supplementary file 1 [file antioxidants-08-00507-s001.zip › Supplementary Files/Supplementary File S3.docx]
