# Supplementary material for: Systematic Physical Exercise and Spirulina maxima Supplementation Improve Body Composition, Cardiorespiratory Fitness, and Blood Lipid Profile: Correlations of a Randomized Double-Blind Controlled Trial
Source: Antioxidants (Basel). 2019 Oct 23;8(11):507. doi: 10.3390/antiox8110507 (PMC6912262; doi:10.3390/antiox8110507)
Supplement: Supplementary file 1 [file antioxidants-08-00507-s001.zip › Supplementary Files/Supplementary File S2.doc]

**Supplementary File 2.** CONSORT 2010 Flow Diagram of the progress through the phases of the trial.

**Enrollment**

**Allocation**

**Allocation**

**Follow-Up**

**Analysis**

Allocated to *Spirulina maxima* intervention (n= 26)

♦ Received allocated intervention (n= 26)

♦ Did not receive allocated intervention (n= 0)

Allocated to placebo intervention (n= 26)

♦ Received allocated intervention (n= 26)

♦ Did not receive allocated intervention (n= 0)

Allocated to placebo intervention (n= 26)

♦ Received allocated intervention (n= 26)

♦ Did not receive allocated intervention (n= 0)

Lost to follow-up (n= 0)	Discontinued intervention (n= 0)

Allocated to *Spirulina maxima* intervention (n= 26)

♦ Received allocated intervention (n=26)

♦ Did not receive allocated intervention (n= 0)

Analysed (n= 52)
♦ Excluded from analysis (n= 0)

Assessed for eligibility (n= 64)

Excluded (n= 12)

♦  Not meeting inclusion criteria (n= 7)

♦  Declined to participate (n= 5)

♦  Other reasons (n= 0)

Randomized (n= 52)
